# Effect of metal decoration on sulfur-based gas molecules adsorption on phosphorene

**DOI:** 10.1038/s41598-021-97626-4

**Published:** 2021-09-13

**Authors:** Yonghu Wang, Shuangying Lei, Ran Gao, Xiaolong Sun, Jie Chen

**Affiliations:** Key Laboratory of Microelectromechanical Systems of the Ministry of Education, Southeast University, Nanjing, 210096 China

**Keywords:** Theory and computation, Nanoscale materials, Electronic properties and materials, Two-dimensional materials

## Abstract

Based on first-principles calculation, the adsorption of sulfur-based gas molecules (H_2_S, SO_2_, SO_3_) on various metal-decorated phosphorenes is researched systematically. Eleven metals (Li, Na, K, Rb, Cs, Ca, Sr, Ba, Ni, La, Tl) which can avoid the formation of clusters on the phosphorene are considered. Noticeably, all metal decorations can enhance the adsorption strength of phosphorene to sulfur-based gas molecules except for H_2_S on Tl-decorated phosphorene. Meanwhile, the adsorption energy (*E*_ads_) shows the trend of *E*_ads_(H_2_S) < *E*_ads_(SO_2_) < *E*_ads_(SO_3_) for the same metal decoration case. In addition, some metal-decorated phosphorene systems exhibit intriguing magnetic and electrical variation after sulfur-based gas molecule adsorptions, indicating that these systems are promising to be candidates for the detection and removal of sulfur-based gas molecules.

## Introduction

Air pollution is becoming more and more serious with the rapid development of industrialization. Thanks to attenuation of organic substances, emission of sewage plants and burning of fossil fuels^1–4^, every year more than billion tons of sulfur-based gases are discharged into the atmosphere^5,6^. The sulfur-containing gas compounds are all dangers for human-health^7–9^. SO_2_ hurts the nerves in the respiratory system, including lesions in nasal cavity and throat. H_2_S inhibits the metabolism of cells in the livers^10,11^. In terms of environmental pollution, sulfur-containing gas compounds can bring about sulfuric acid mist, sulphate aerosol, as well as acidic soil, which further harm animals and green plants^12,13^. Therefore, the treatment of sulfur-based exhaust gases is essential in environmental safety.

Adsorption is great competitive in the removal and detection of sulfur-based gases. A lot of experimental and theoretical investigations on the adsorption of sulfur-based gas compounds on metal and metal oxide are performed^14–16^. However, owing to the strict operating condition and low sensitivity, the metal and metal oxide are not the ideal candidate materials for sensing the sulfur-based gas compounds^17^. On the other hand, since the discovery of graphene in 2004^18–20^, two-dimensional (2D) materials have aroused great interest of researchers owing to their superior mechanical, thermal, optical and electronic properties^21–24^. Another striking feature of 2D materials is the large surface-to-volume ratio, which may be attractive for gas detection and adsorption. Recently, the researchers observed that phosphorene has an advantage over graphene on the adsorption of small molecule gases because of its puckered surface morphology and higher surface-to-volume ratio^25,26^. Both theoretical and experimental researches have proven excellent gas sensing sensitivity of phosphorene^27,28^. However, the *E*_*ads*_ values of gases on pristine phosphorenes are too small, hence the surface decoration or/and doping is demanded to enhance the adsorption of gases^29–31^. For sulfur-based gas adsorption on phosphorene, the adsorptions of defective and metal substitute doped phosphorenes to H_2_S and SO_2_ were investigated by Kaewmaraya, which exposed that metal-dopings could significantly enhance the adsorption of phosphorene to SO_2_^32^.

In this investigation, we have systematically studied the adsorption of sulfur-based gas molecules (H_2_S, SO_2_, SO_3_) on various metal-decorated phosphorene by using the first-principles calculation. To avoid clustering of metals on the surface of phosphorene, eleven metals have been opted on the basis of their bulk cohesive energy less than the binding energy. They are alkali (Li, Na, K, Rb, Cs), alkaline earth (Cs, Ca, Sr), transition (Ni, La), and post-transition (Tl) metals. Except for H_2_S on Tl-decorated phosphorene, all metal decorations can improve the adsorption of sulfur-based gas molecules on phosphorene, especially Ni and Tl-decorated phosphorene. In addition, some metal-decorated phosphorene systems exhibit interesting magnetism and electrical transitions after SO_2_ and SO_3_ gas adsorption, which could have potential application for SO_2_ and SO_3_ gas detection.

## Computational methods

In this article, all the density functional theory (DFT) calculations have been carried out by the Vienna ab initio simulation software package code (VASP)^33,34^. We took advantage of the Perdew-Burke-Ernzerhof (PBE) functional of generalized gradient approximation (GGA) to describe exchange–correlation interaction^35^. The van der Waals (vdW) interactions were dealt with by adopting empirical correction scheme of Grimme (DFT + D3)^36^. In all the calculations, the kinetic energy cut-off for the plane-wave basis was 500 eV. The 3 × 4 supercells and the vacuum distances of 15 Å were utilized to reduce the interaction of mirror adsorbates and phosphorene layers, respectively, and the corresponding *k*-point grids were set as 3 × 3 × 1 by Monkhorst–Pack k-point scheme. The energy convergence accuracy was set to 10^−5^ eV, and all the structures were fully relaxed until the forces acting upon each atom were less than 0.01 eV/Å. For sulfur-based gas molecules adsorption on pristine phosphorene or metal-decorated phosphorene, the *E*_ads_ is calculated by the formula,

1 $${E}_{ads}={E}_{tot}-\left({E}_{sub}+{E}_{gas}\right),$$

where ${E}_{tot}$, ${E}_{sub}$ and ${E}_{gas}$ are the energies of the adsorption system, substrate (pristine phosphorene or metal decorated phosphorene) and sulfur-based gas molecule (H_2_S, SO_2_, or SO_3_), respectively.

## Results and discussion

### Sulfur-based gas molecules adsorption on pristine phosphorene

**Figure 1:**
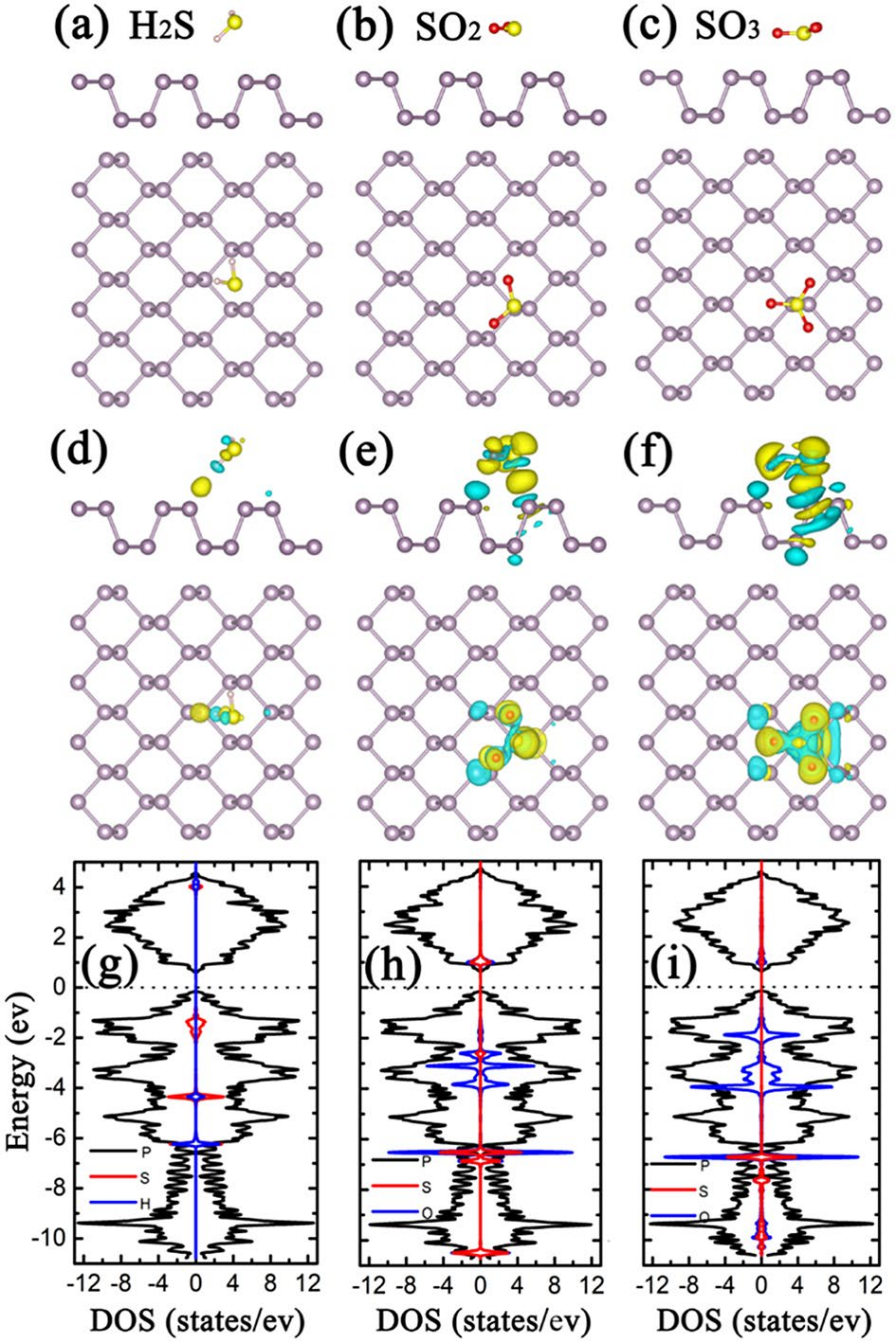
The optimized structure of (**a**) H_2_S, (**b**) SO_2_ and (**c**) SO_3_ adsorbed 3 × 4 pristine phosphorene, (**d**–**f**) the DCD corresponding to (**a**–**c**), respectively, (**g**–**i**) the LDOS corresponding to (**a**–**c**), respectively. Purple, yellow, pink, and red balls in (**a**–**f**) represent P, S, H and O atoms, respectively. Yellow and blue regions in (**d**–**f**) denote charge accumulation and charge depletion, respectively. The black, red and blue curves in (**g**–**i**) represent LDOS of P, S and H (O), respectively, with the Fermi level set to zero.

The lattice constants of the pristine phosphorene monolayer along the armchair and zigzag directions are 4.57 and 3.31 Å, respectively, with a direct bandgap of 0.88 eV, in agreement with previous studies^37,38^. For the adsorption of sulfur-based gas molecules (H_2_S, SO_2_, SO_3_) on pristine phosphorene, various possible initial adsorption sites [e.g. hollow (H), bridge (B), top (T)] and adsorption configurations were considered [Figures S1–S3]. By comparing the total energies of the adsorption configurations after structural optimization, the configurations with the highest *E*_ads_ were obtained as shown in Fig. 1. The H_2_S prefers to adsorb at the hollow site with H atoms pointing to the phosphorene surface, while the SO_2_ and SO_3_ prefer to adsorb at the T site lying parallel to the phosphorene surface. Such a phenomenon may be attributed to the larger electronegativity of O compared with H. The nearest atom-to-atom distances between H_2_S, SO_2_ and SO_3_ and the surface of the phosphorene are 2.818, 2.984 and 2.572 Å, respectively. The *E*_*ads*_ values of H_2_S, SO_2_ and SO_3_ on phosphorene, calculated using Eq. (1), are 0.220, 0.396 and 0.646 eV, respectively. To analyze the mechanism of interaction between sulfur-based gas molecules and phosphorene, the differential charge density (DCD) of the most stable adsorption configurations were calculated, as illustrated in Fig. 1d–f. It can be seen that, from H_2_S to SO_2_ to SO_3_, the electron accumulations around gas molecules increase significantly, which are also confirmed by the Bader charge analysis. The Bader charge analysis shows that the electrons transferred from the phosphorene to H_2_S, SO_2_ and SO_3_ molecules are 0.003, 0.182 and 0.451 e, respectively, ascribing to higher electronegativity of gas molecules over the phosphorene. Generally, the more charge quantities transferred means the stronger interactions, and thus the larger *E*_*ads*_ values^39^. Here, the variation trend of charge quantities transferred does agree with that of the *E*_*ads*_ values (see Table 1).

**Table 1:** The *E*_*ads*_ values of sulfur-based gas on phosphorene and bP-Ms.

Metal	*E*_*ads*_ (eV)			*∆Q* (e)			*D*_*sub-gas*_ (Å)		
	H_2_S	SO_2_	SO_3_	H_2_S	SO_2_	SO_3_	H_2_S	SO_2_	SO_3_
bP	0.220	0.396	0.646	− 0.003	− 0.182	− 0.451	2.818	2.984	2.572
Li	0.695	1.213	1.684	− 0.080	− 0.626	− 1.004	2.484	2.003	1.952
Na	0.441	1.121	1.208	− 0.011	− 0.523	− 0.806	2.875	2.374	2.286
K	0.287	0.945	0.979	0.026	− 0.422	− 0.674	3.399	2.799	2.673
Rb	0.251	0.949	0.716	0.022	− 0.449	− 0.526	3.610	2.978	2.640
Cs	0.208	0.810	0.828	0.019	− 0.399	− 0.640	3.844	3.182	3.001
Ca	0.584	2.086	3.657	0.014	− 0.862	− 1.416	2.963	2.263	2.147
Sr	0.485	1.929	3.419	0.010	− 0.830	− 1.435	3.193	2.439	2.295
Ba	0.686	1.657	3.232	− 0.059	− 0.801	− 1.484	3.307	2.633	2.591
Ni	1.158	1.304	1.605	0.042	− 0.318	− 0.724	2.207	2.074	1.977
La	0.773	2.822	4.508	− 0.044	− 1.157	− 1.325	3.136	2.272	2.175
Tl	0.136	0.597	1.273	0.021	− 0.418	− 0.832	3.795	2.978	2.721

The *∆Q* and *D*_*sub-gas*_ between sulfur-based gas and substrates.

To understand the effect of the adsorption of sulfur-based gas molecules on the electrical properties of phosphorene, the local density of states (LDOS) of the adsorption system were calculated, as shown in Fig. 1. In LDOSs, the DOS near the Fermi level is zero for all three adsorption systems, indicating the adsorption of sulfur-based gas don’t change the phosphorene’s electronic structure. The bandgaps of phosphorenes are 0.86 and 0.87 eV, respectively, after H_2_S and SO_2_ adsorptions, which are marginally smaller than that of pristine one, while the bandgap of phosphorene slightly increase to 0.89 eV after SO_3_ adsorption. After sulfur-based gas molecule adsorptions, the slight variation of bandgap may be ascribed to the change of channel of phosphorene. It has been pointed out that a narrower channel (3.49 Å for H_2_S and 3.44 Å for SO_2_ as compared to 3.54 Å for pristine phosphorene) would result in the stronger repulsive interaction between the facing lone pairs at the ditch of phosphorene and thus the decrease of bandgap, and vice-versa^40^. For H_2_S adsorption case, the H atom is the nearest to the P atom, but the H atomic DOS is far from the Fermi level, which should be also responsible for the smaller *E*_*ads*_ value. In the case of SO_2_ and SO_3_, the S atoms are the closest to P atoms. Near Fermi levels, the S DOS peak is just above the conduction band minimum. Especially for SO_3,_ the S DOS distributes widely in the conduction band, which should be responsible for the largest *E*_*ads*_ values among the three adsorption cases. The adsorption energy closed to 1 eV is an ideal binding for the efficient and reversible gas sensor. However, the *E*_*ads*_ values of sulfur-based gas molecules on pristine phosphorene are too small for this purpose. The metal decoration can improve the adsorption of gas, so we will discuss the metal decorations and the sulfur-based gas molecule adsorptions on metal decorated phosphorenes.

### Sulfur-based gas molecules adsorption on bP-Ms

**Figure 2:**
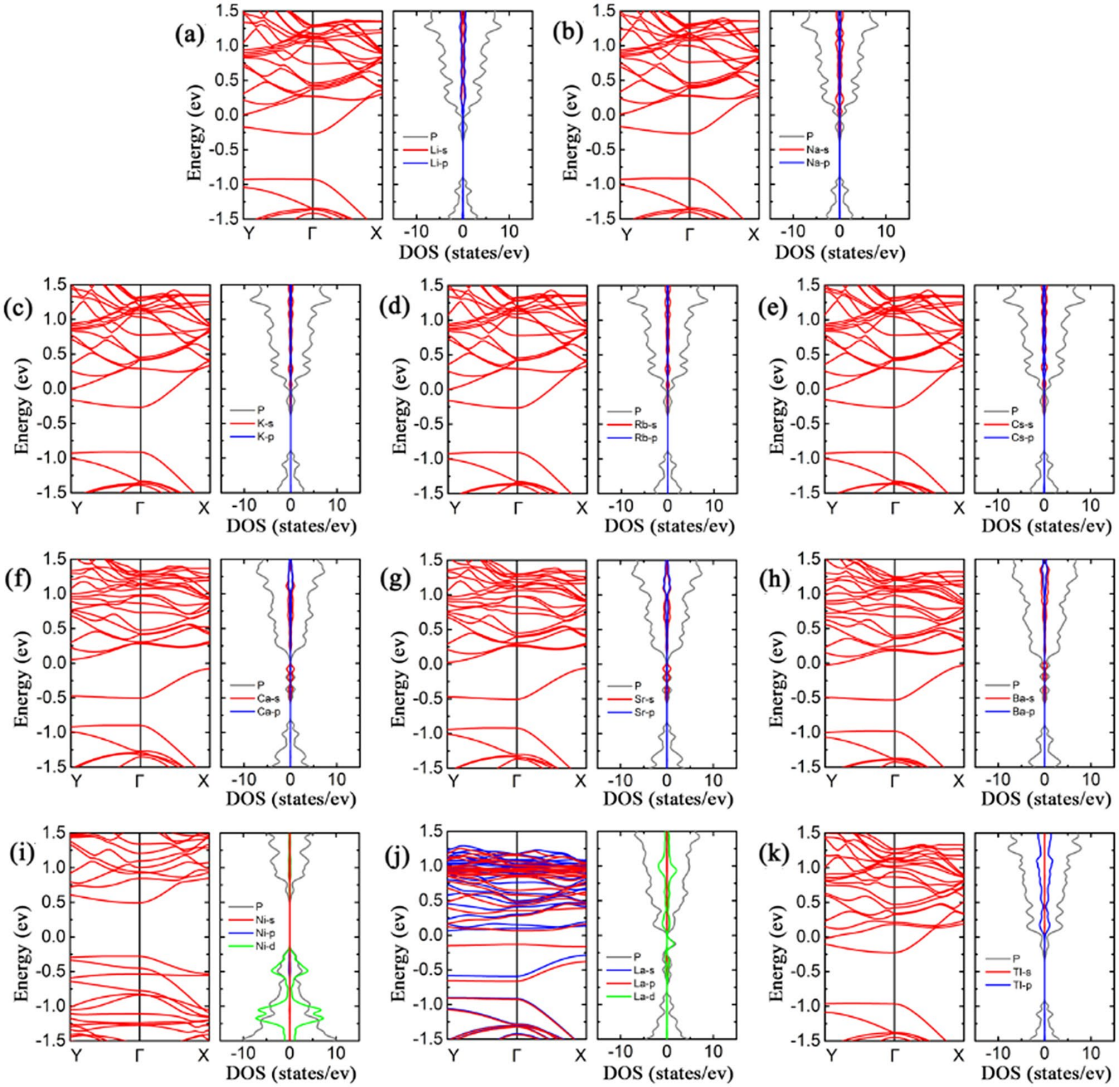
The band structures and projected DOSs of (**a**) bP-Li, (**b**) bP-Na, (**c**) bP-K, (**d**) bP-Rb, (**e**) bP-Cs, (**f**) bP-Ca, (**g**) bP-Sr, (**h**) bP-Ba, (**i**) bP-Ni, (**j**) bP-La and (**k**) bP-Tl. The red and blue curves represent the spin-up and spin-down bands, respectively. The gray curves in projected DOS represent state of P, and the red, blue and green curves represent the *s-*, *p-* and *d*-states for metal atoms.

The theoretical studies have shown that metal decoration can significantly influence the electronic properties of phosphorene^41^. It is well-known if the *E*_*ads*_ value of a metal on 2D materials is less than its bulk cohesive energy (*E*_coh_), it is going to cluster on the 2D surfaces. Therefore, to enhance the adsorption of sulfur-based gas on phosphorenes and avert clustering of metal atoms on the surface, eleven metals (Li, Na, K, Rb, Cs, Ca, Sr, Ba, Ni, La, Tl) have been opted to decorate the phosphorene on the basis of *E*_ads_/*E*_coh_ > 1^42^. Here, the electronic properties of black phosphorenes decorated with metals (bP-Ms) are discussed first to contrast before and after sulfur-based gas molecule adsorptions. Figure 2 shows the band structures and projected density of states (PDOSs) of the bP-Ms. Except for Ni, the outermost *s*-states of alkali metals (AMs = Li, Na, K, Rb, Cs) and those of alkaline earth metals (AEMs = Ca, Sr, Ba), the 6 *p*-state of Tl and 5 *d*-state of La are mainly distributed in the conduction band of phosphorenes, which cause valence-electron transfer from metals to phosphorenes, and thus the Fermi levels shift up in energy. For AM decorated and Tl decorated phosphorenes (bP-AMs and bP-Tl), the Fermi levels shift upward into the conduction bands of phosphorenes and the corresponding bP-AMs and bP-Tl show metal properties. The *s*-states of AMs and *p*-state of Tl are far above the lowest conduction bands of phosphorenes. Unlike the AMs and Tl, the *s*-states of AEMs or *d*-state of La are aligned to the lowest conduction band of phosphorene, which causes a strongly repulsive interaction between metal atomic states and the lowest conduction bands of phosphorenes, so the lowest conduction bands are pushed down. As a consequence, there are large separations between the lowest and second lowest conduction bands for AEM decorated phosphorenes (bP-AEMs) and bP-La. On the other hand, the more *s*- and *d*-electrons transfer for bP-AEMs and bP-La, the more energies shift for Fermi levels. The Fermi levels of bP-AEMs and bP-La are located between the lowest and second lowest conduction bands. The bP-Ca, bP-Sr and bP-La show semiconductor properties, while bP-Ba shows metal property due to that the Fermi level cross slightly through the second lowest conduction band of phosphorene. Additionally, bP-La has spin-polarized LDOS (Fig. 2), with magnetic moment of 1 ${\upmu }_{\mathrm{B}}$. For Ni decorated case, the *s*- and *d*- states are distributed in valence band of phosphorene, and the semiconductor property of bP-Ni is remained. However, the bandgap of bP-Ni decreases to 0.769 eV as compared with that of pristine phosphorene, which may be ascribed to the strong hybridized interaction between Ni *d*-state and valence band of phosphorene, and thus the highest valence band is pushed upward.

### H_2_S gas molecules adsorption on bP-Ms

**Figure 3:**
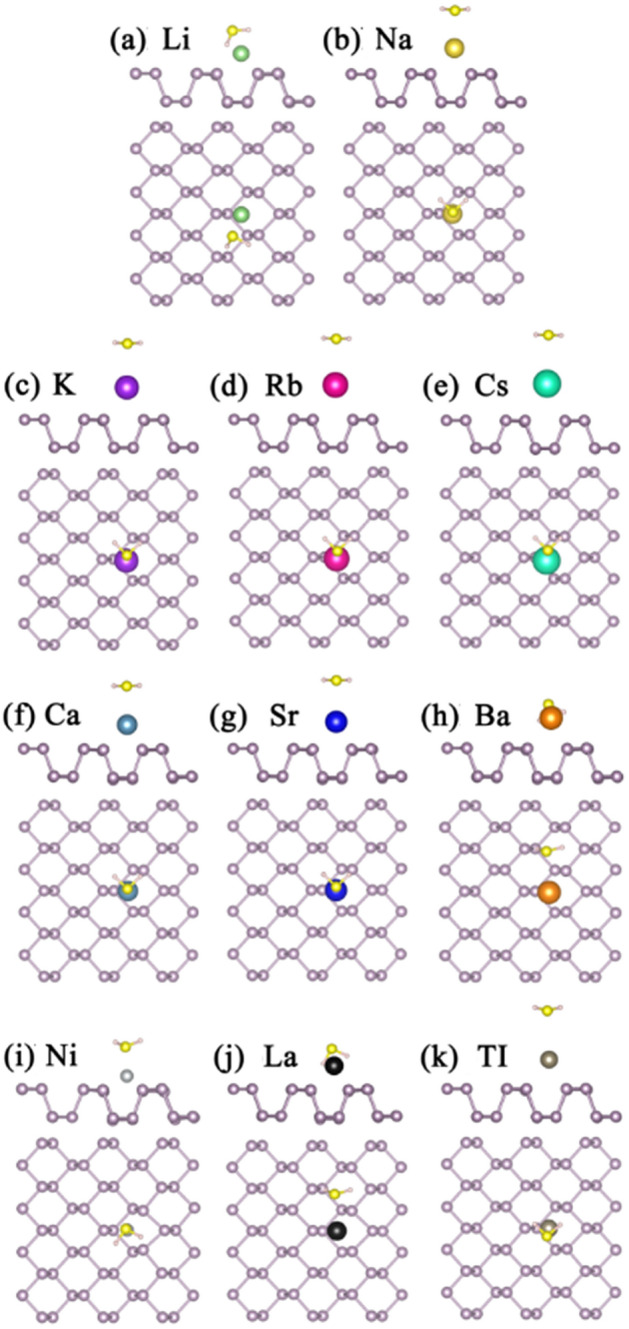
The optimized structures of H_2_S adsorption on (**a**) bP-Li (**b**) bP-Na (**c**) bP-K (**d**) bP-Rb (**e**) bP-Cs (**f**) bP-Ca (**g**) bP-Sr (**h**) bP-Ba (**i**) bP-Ni (**j**) bP-La (k) bP-Tl. The green, gold, purple, red, cyan, dark cyan, blue, orange, light gray, black and gray balls represent Li, Na, K, Rb, Cs, Ca, Sr, Ba, Ni, La and Tl, respectively.

For the H_2_S adsorption on bP-Ms (M = Li, Na, K, Rb, Cs, Ca, Sr, Ba, Ni, La, Tl), the various initial configurations are considered and are fully optimized. By comparing the *E*_*ads*_ values, the most stable structures, namely the structures with the largest absolute *E*_*ads*_ values, are achieved as shown in Fig. 3. In all the structures, the distances between the sulfur and metal atoms are apparently nearer than those between H and metal atoms. Except for bP-Li, bP-Ba and bP-La systems, H_2_S is adsorbed on the bP-Ms in parallel to the surface of phosphorene. The *E*_*ads*_ values of S-based gases, the charge transfer amounts (*∆Q*) and adsorption distances (*D*_*sub-gas*_) between sulfur-based gas molecules and substrates are summarized in Table 1. As compared with the adsorption energy of 0.220 eV for H_2_S on pristine phosphorene, except for Cs and Tl atoms, the decorations of the rest metals enhance the adsorption of phosphorene to H_2_S. Especially, the *E*_*ads*_ value of H_2_S on bP-Ni is up to 1.158 eV (see Table 1), which is the largest value for all the H_2_S adsorption systems. Correspondingly, the adsorption distance between Ni and S atoms is the smallest among the adsorption of H_2_S on the metal decorated phosphorene, and the value is 2.207 Å. However, the adsorption energies of H_2_S on bP-Tl and bP-Cs are the smallest, even smaller than that on pristine phosphorene. On the one hand, it may be due to the fact that the electrons of all atoms in H2S molecule are full shell structure, resulting in less charge transfer and interaction; On the other hand, it may be attributed to the relatively large atomic radii of Tl and Cs, which cause the longer adsorption distance as well as less interaction between gas and metal decorated phosphorene. The H_2_S on bP-Tl and bP-Cs have the largest adsorption distance of 3.795 and 3.844 Å, respectively, which may be due to the larger Tl atomic size.

**Figure 4:**
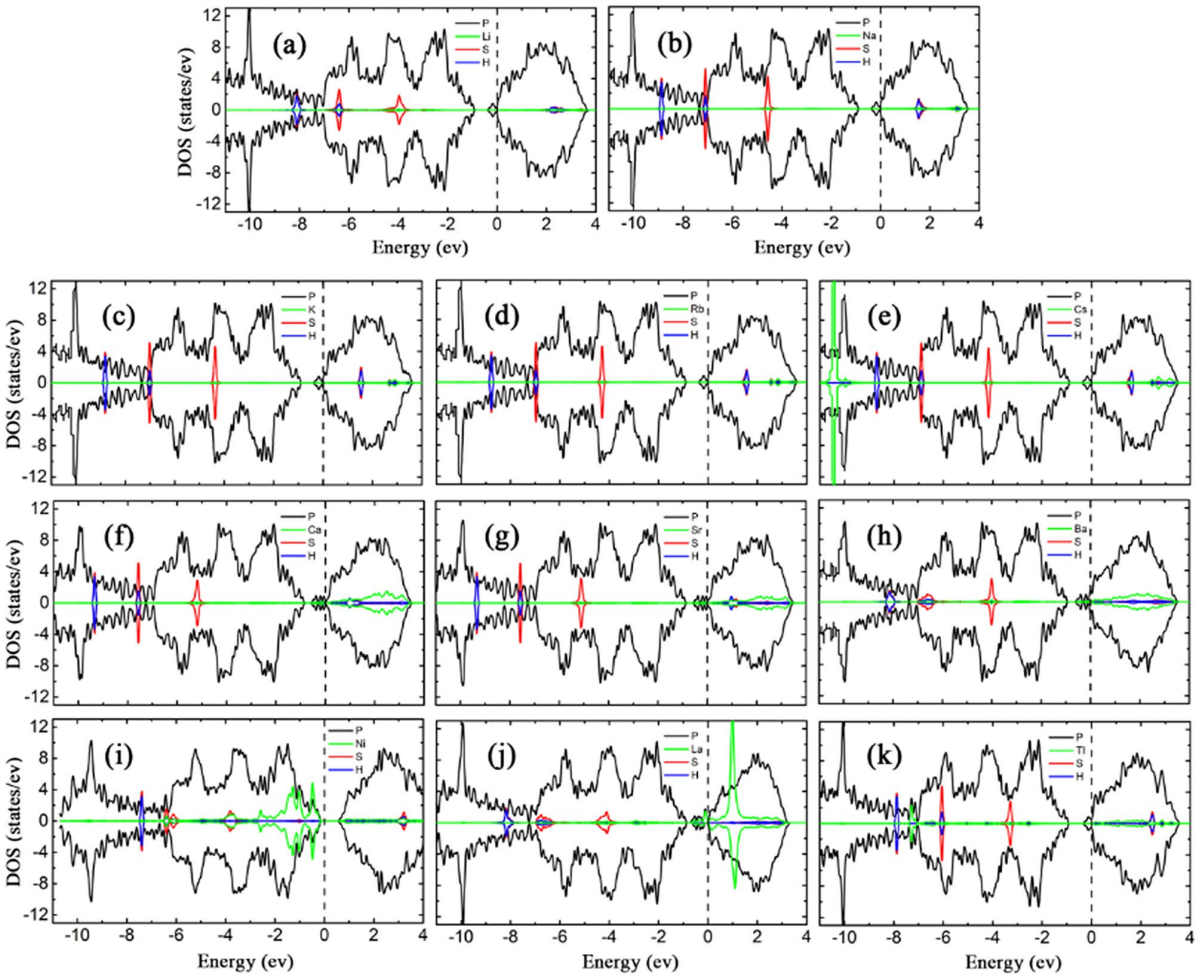
The LDOSs of H_2_S adsorption on (**a**) bP-Li (**b**) bP-Na (**c**) bP-K (**d**) bP-Rb (**e**) bP-Cs (**f**) bP-Ca (**g**) bP-Sr (**h**) bP-Ba (**i**) bP-Ni (**j**) bP-La (k) bP-Tl. The black, green, red and blue curves represent LDOS of P, metal, S and H, respectively, with the Fermi level set to zero.

To understand the mechanism of adsorption-energy enhancement and the effect of H_2_S on the electronic and magnetic properties of bP-AM, the LDOSs of the adsorption systems were calculated, as shown in Fig. 4. The magnetic moment of bP-La reduces slightly from 1.00 to 0.98 ${\upmu }_{\mathrm{B}}$ after H_2_S adsorption, while those of the other bP-Ms systems remain zero, which are corroborated by the spin asymmetric LDOS for H_2_S adsorbed bP-La and the spin symmetric LDOSs for the other H_2_S adsorbed bP-M systems. Except for Ca, Sr and La decorated cases, the adsorptions of H_2_S have no effect on the electronic properties of the rest bP-M systems. The metal or semiconductor properties of the rest bP-M systems remained after H_2_S adsorption. The adsorption of H_2_S enhances the interaction between Ca/Sr atom and the conduction band of phosphorene, which leads to a slight downward shift of the conduction band and the Fermi energy level entering the sub-low conduction band, and further causes that bP-Ca and bP-Sr undergo the transformation from semiconductor to metal after the adsorption of H_2_S molecule. As shown in Fig. 4, there are the overlapping peaks between the sulfur and metal atoms located near − 5 or − 4 eV for AMs, AEMs and La decorated cases, which should be a reason of the improved H_2_S adsorption. The smallest *E*_*ads*_ values for Cs and Tl decorated may be mainly attributed to the large radii of Cs and Tl atoms. For Ni decorated case, the apparent overlapping peaks of S and Ni atoms near − 6, − 4 and − 1 eV imply the strong hybridized interaction between H_2_S and bP-Ni substrate, which may be primarily responsible for the largest *E*_*ads*_ value. Additionally, the states of S and H atoms are far from the Fermi level, implying that H_2_S adsorption almost has no effect on the band structures near it for bP-Ms. Thus, the conductivity properties of bP-Ms are not changed by H_2_S adsorption except for Sr and La decorated cases. For Sr decorated case, the state of H_2_S molecule in conduction band pushes slightly the conduction band minimum of bP-Sr down, which makes the small bandgap (0.07 eV) of bP-Sr disappear. For La decorated case, the charge transfer from substrate to H_2_S makes the Fermi level shift down in energy after H_2_S adsorption, and finally the Fermi level crosses through the spin-up band [see the red curve below the Fermi level in Fig. 2(j)], which leads to the half-metal property of bP-La.

### SO_2_ gas molecules adsorption on bP-Ms

**Figure 5:**
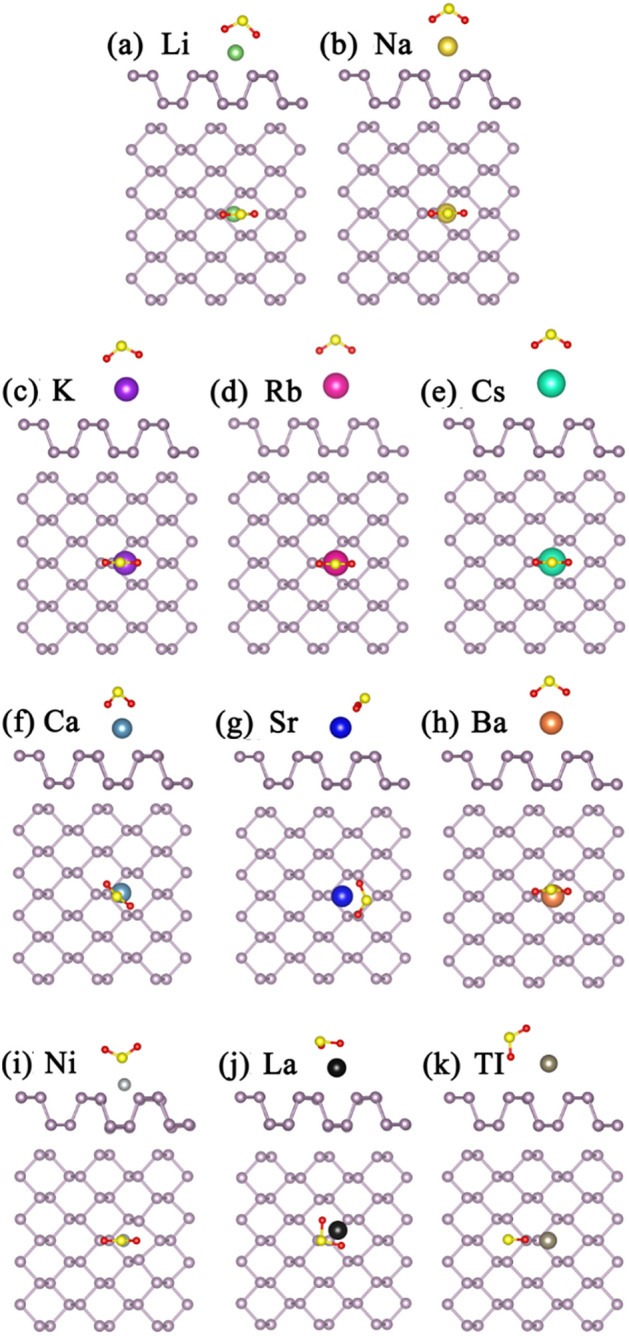
The optimized structures of SO_2_ adsorption on (**a**) bP-Li (**b**) bP-Na (**c**) bP-K (**d**) bP-Rb (**e**) bP-Cs (**f**) bP-Ca (**g**) bP-Sr (**h**) bP-Ba (**i**) bP-Ni (**j**) bP-La (k) bP-Tl. The green, gold, purple, red, cyan, dark cyan, bule, orange, light gray, black and gray balls represent Li, Na, K, Rb, Cs, Ca, Sr, Ba, Ni, La and Tl, respectively.

For the SO_2_ adsorption on bP-Ms, the most stable structures are illustrated in Fig. 5. Except for SO_2_ adsorption on bP-Ni, the SO_2_ preferably adsorbs on the bP-AMs with bond angles toward the metal atoms, and oxygens of SO_2_ are the nearest atoms to the metal atoms. As given in Table 1, all the metal decorations can significantly improve the adsorption capacity of phosphorene to SO_2_. The corresponding *E*_*ads*_ values vary from 0.597 to 2.822 eV, and stay larger than the pristine phosphorene case of 0.396 eV. In addition, we can see that the decoration of AEMs (Ca, Sr, Ba) is more effective than AMs (Li, Na, K, Rb, Cs) for adsorbing SO_2_ due to larger *E*_*ads*_ values (Table 1), and that decrease with atomic number in the same group elements. As we know, the more charge transfer means the stronger interaction, i.e. larger *E*_*ads*_ value. Consequently, the more valence electrons of AEMs and thus the more electron transfers should be in charge of their larger *E*_*ads*_ values. On the other hand, short adsorption distance is beneficial for the charge transfer. Thus, an increasing adsorption distance should be responsible for decreasing *E*_*ads*_ values in the same group elements. Adsorption distance may be attributed to the atomic radius increasing with atomic number.

**Figure 6:**
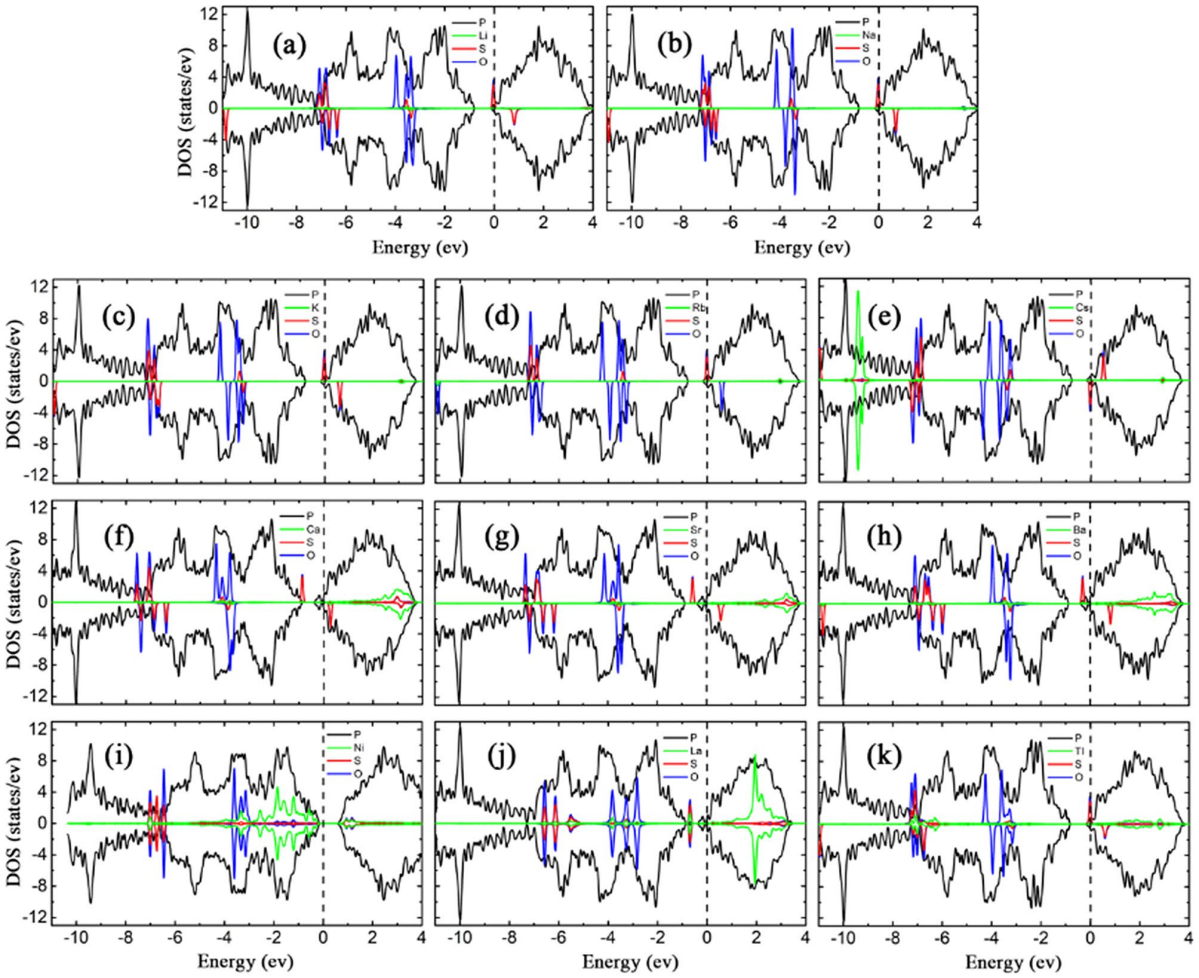
The LDOSs of SO_2_ adsorption on (**a**) bP-Li (**b**) bP-Na (**c**) bP-K (**d**) bP-Rb (**e**) bP-Cs (**f**) bP-Ca (**g**) bP-Sr (**h**) bP-Ba (**i**) bP-Ni (**j**) bP-La (k) bP-Tl. The black, green, red and blue curves represent the LDOS of P, metal, S and O, respectively, with the Fermi level set to zero.

To understand how the SO_2_ effect on the properties of the metal decorated substrates, the LDOSs of all SO_2_ adsorption systems were calculated and shown in Fig. 6. The non-zero LDOSs at the Fermi level imply the metal properties for SO_2_ adsorptions on bP-AM, bP-AEM, bP-La and bP-Tl systems, while the zero LDOSs at the Fermi level imply semiconductor properties for that on bP-Ni system. Except for bP-Ni, almost all the bP-Ms exhibit spin asymmetry after SO_2_ adsorption. The SO_2_ adsorbed bP-La has a negligible magnetic moment of 0.0002 µ_B_, on the spin polarization of bP-La system is suppressed by the SO_2_ adsorption. Thus, the bP-AMs, bP-AEMs and bP-Tl perceive magnetization on SO_2_ adsorption, while bP-La loses the magnetization. In addition, the bP-Ca, bP-Sr and bP-La become metallic, on upward movement of the lowest conduction band of phosphorene (see figure S6 in supporting materials). In fact, upward movements of the lowest conduction bands exist in all the SO_2_ adsorbed bP-M systems, including the SO_2_ adsorption on bP-AM, bP-Ba and bP-Tl systems (although their metal properties remained), which may be attributed to that the SO_2_ adsorptions weaken the interactions between the metals and phosphorenes. For SO_2_ adsorption on bP-Ni, upward movement of the lowest conduction band results in that the bandgap of bP-Ni rises from 0.769 to 0.876 eV after SO_2_ adsorption. On the other hand, as compared with before SO_2_ adsorption, the electron transfers from bP-M systems to SO_2_ lead to downward shifts of the Fermi levels for bP-M systems, which may be ascribed to the greater electronegativity of S and O atoms. Except for Ni decorated case, the peaks of O states appear near the Fermi levels, which will introduce the flat bands into the band structures for SO_2_ adsorption on bP-Ms (see figure S6). As compared with SO_2_ adsorption on the AM systems, the coupling peaks between SO_2_ and AEMs are more widely distributed, especially in the conduction band (Fig. 6), which may be a reason of more effectively improving SO_2_ adsorption on phosphorene for the AEM decorated cases. Strong hybridized interaction between the states of SO_2_ and La at − 0.8 eV in Fig. 6j can unravel a maximal *E*_*ads*_ = 2.822 eV for SO_2_ adsorption on bP-La.

### SO_3_ gas molecules adsorption on bP-Ms

**Figure 7:**
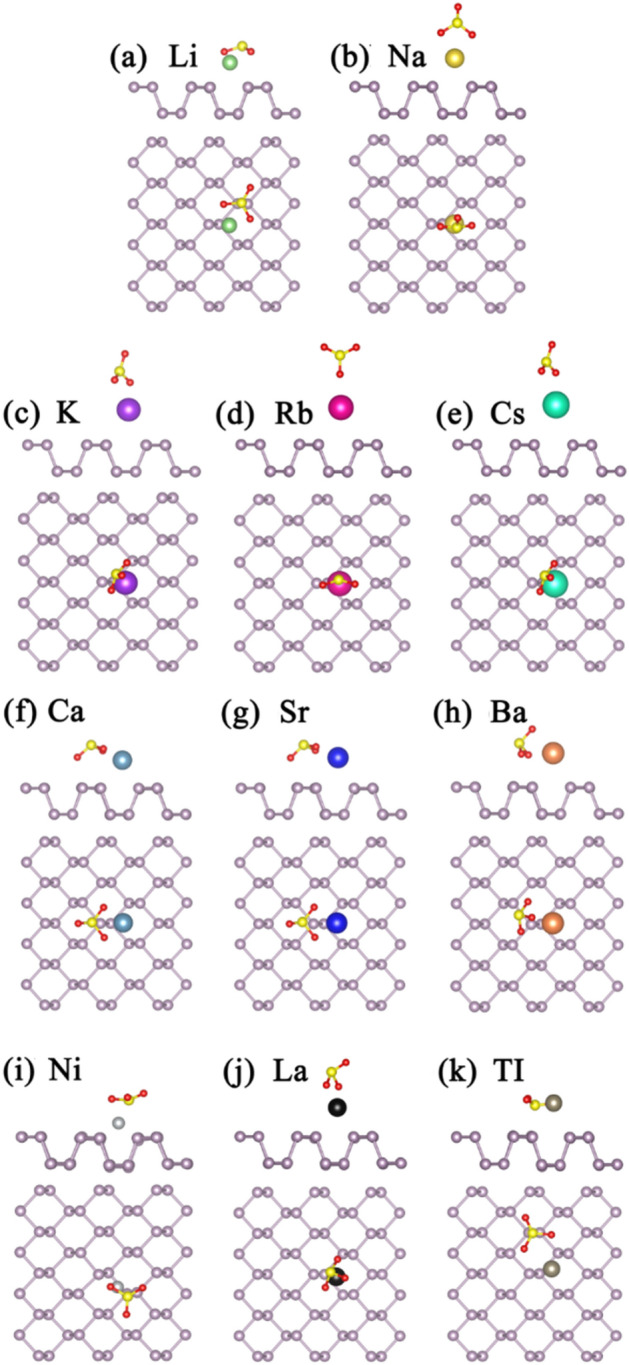
The optimized structures of SO_3_ adsorption on (**a**) bP-Li (**b**) bP-Na (**c**) bP-K (**d**) bP-Rb (**e**) bP-Cs (**f**) bP-Ca (**g**) bP-Sr (**h**) bP-Ba (**i**) bP-Ni (**j**) bP-La (k) bP-Tl. The green, gold, purple, red, cyan, dark cyan, bule, orange, light gray, black and gray balls represent Li, Na, K, Rb, Cs, Ca, Sr, Ba, Ni, La and Tl , respectively.

Optimized structures of SO_3_ adsorption on bP-Ms in different initial configurations are shown in Fig. 7. Compared to H_2_S and SO_2_, the most stable adsorption configurations of SO_3_ on bP-Ms are more diverse and have larger *E*_*ads*_ values for the same metal decorated substrate. However, in all the adsorption configurations of SO_3_ on bP-Ms, the O in SO_3_ is the nearest to metal atoms. The corresponding adsorption energies of 0.716 to 4.508 eV (Table 1) are larger than the 0.646 eV value of SO_3_ on pristine phosphorene. Especially, the adsorption configuration of SO_3_ on bP-Tl is similar to that on pristine phosphorene, but the *E*_*ads*_ value increases from above value of pristine phosphorene to 1.273 eV due to the Tl atomic decoration. Thus, all the metal decorations significantly improve the SO_3_ adsorption on phosphorene. Similar to SO_2_, *E*_*ads*_ values of SO_3_ on bP-AEM are larger than that on bP-AM, indicating more efficient decorations of AEMs than AMs in improving the SO_3_ adsorption on phosphorene. As mentioned above, the more charge transfer signifies the stronger interaction and in turn the larger *E*_*ads*_ value^39^. Consequently, the more valence electrons of AEMs and thus electron transfers should be responsible for their larger *E*_*ads*_ values. On the other hand, in the same group elements, the *E*_*ads*_ values decrease with the atomic number in lieu of increasing atomic size. As mentioned before, short adsorption distance is advantageous to the charge transfer^26,27^. The larger atomic size means the larger adsorption distance and thus the more charge transfer, leading to larger interaction and larger *E*_*ads*_ value. Additionally, we can see (Table 1) the electrons transfer from bP-Ms to SO_3_ in SO_3_ adsorbed bP-Ms, in accords to larger S and O electronegativities.

**Figure 8:**
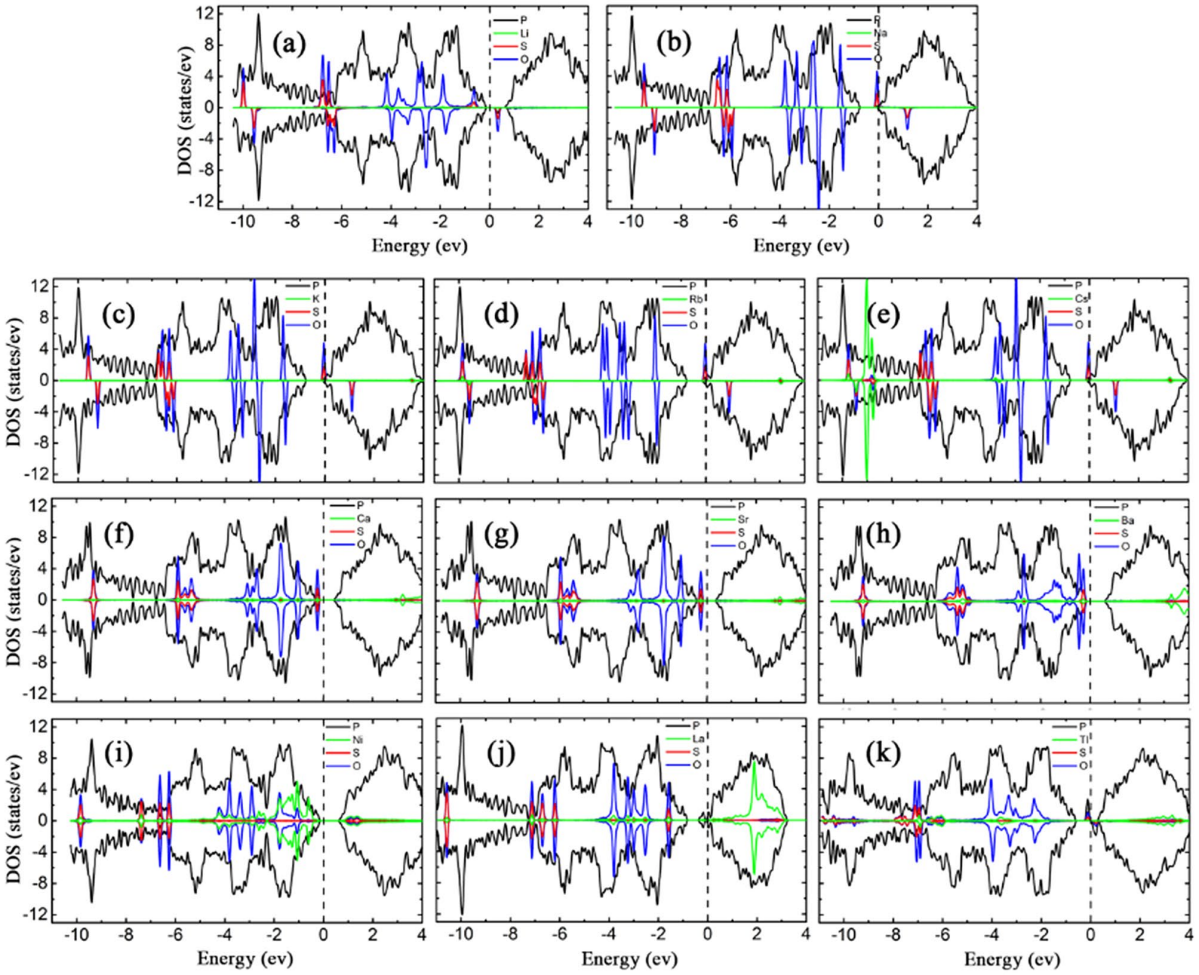
The LDOSs of SO_3_ adsorption on (**a**) bP-Li (**b**) bP-Na (**c**) bP-K (**d**) bP-Rb (**e**) bP-Cs (**f**) bP-Ca (**g**) bP-Sr (**h**) bP-Ba (**i**) bP-Ni (**j**) bP-La (k) bP-Tl. The black, green, red and blue curves represent the LDOS of P, metal, S and O, respectively, with the Fermi level set to zero.

In Fig. 8, LDOSs of SO_3_ adsorbed bP-Ms display marked effects of SO_3_ on the bP-Ms substrates. The LDOSs of bP-AMs and bP-Tl reveal significant spin asymmetry after SO_3_ adsorptions, which are duly induced by SO_3_, indicating magnetism in the SO_3_ adsorbed bP-AMs and bP-Tl. The SO_3_ adsorbed bP-AEMs and bP-Ni have spin symmetrical LDOSs and no magnetism. The magnetic moment of SO_3_ adsorbed bP-La is only 0.0003 µ_B_, which can be ignored with almost spin symmetrical LDOSs. Therefore, the bP-AMs and bP-Tl develop magnetism on SO_3_ adsorption, while the bP-La adversely loses the magnetism. The SO_3_ adsorbed bP-Li, bP-Na, bP-AEMs, bP-Ni and bP-Tl systems have zero DOSs at the Fermi levels of semiconductor properties. The SO_3_ adsorbed bP-K, bP-Rb, bP-Cs and bP-La have non-zero DOSs at the Fermi levels, implying that they possess metal properties. In contrast to before SO_3_ adsorption, the bP-Li, bP-Na, bP-Ba and bP-Tl develop metal-to-semiconductor transitions after SO_3_ adsorption, while the bP-La develops the semiconductor-to-metal transition. As showed in figure S7 of supporting materials, the effect of SO_3_ on the band structures of bP-M systems is the largest among the three gas molecules, likely due to the largest amount of electron transfer from the bP-Ms substrate to SO_3_.

In addition, the SO_3_ introduces impurity states into/near bandgaps or into the valence bands. Accordingly, the Fermi levels of bP-Ms shift down differently as a result of a charge transfer from the substrate to SO_3_. As shown in Fig. 8a,b, the highest spin-up states of SO_3_ for bP-Li and bP-Na systems are located in valence and bandgap, respectively, and both are below the Fermi levels. This leads to more electron transfer from bP-Li and bP-Na to SO_3_, a way in witch bP-Li and bP-Na have restored to be semiconductor, which is responsible for their metal-to-semiconductor transitions. But for bP-K, bP-Rb and bP-Cs systems, as shown in Fig. 8c–e, the highest spin-up SO_3_ states cross through the lowest conduction bands of bP-Ms, leading to less electrons transfer and thus metal properties being remained. For bP-AEM, both the highest spin-up and spin-down bands are located near valence band maximum (VBM) and below the Fermi levels, resulting in that the Fermi levels shift down due to electrons transferring from bP-AEM to SO_3_. On the other hand, the AEM − SO_3_ interaction weakens the corresponding AEM − phosphorene interactions, and thus the lowest conduction band moves upward and the energy separation between the lowest and the second lowest conduction bands restores to pristine phosphorene value. Consequently, the bP-AEMs show semiconductor properties after SO_3_ adsorption, and complete the metal-to-semiconductor transitions. For SO_3_ adsorbed bP-Ni system, the bandgap of bP-Ni system increases to 0.882 eV due to the AEM − SO_3_ interaction. The VBM is transferred from $\Gamma \mathrm{ point}$ to Y point, and thus the bP-Ni exhibits indirect semiconductor properties and experiences a direct-to-indirect transition. For SO_3_ adsorbed bP-La system, the metal flat band disappears and thus the lowest conduction band shifts upward owing to the La − SO_3_ interaction on phosphorene. This leads to the metal → semiconductor transition of the bP-Tl system after SO_3_ adsorption. This may be ascribed to the SO_3_ flat bands in bandgap of bP-Tl, which push upward the lowest conduction band. Compared to the LDOS of SO_3_ adsorbed bP-AM systems, the coupling peaks between SO_3_ − metals are more widely distributed at − 9 eV, − 6 to − 5 eV, − 3 to 0 eV and in the conduction band, which also may be a reason of AEM decoration is more effective in improving the SO_3_ adsorption on phosphorene. For SO_3_ adsorbed bP-Ni, bP-La and bP-Tl systems, there are many coupling peaks between the states of metal and SO_3_ near the Fermi level, which may be responsible for the large interactions between bP-Ms and SO_3_. As compared with other metal cases, more coupling peaks of La and SO_3_ are located in the band, contributing to the excellent SO_3_ adsorption performance on the bP-La surfaces.

The values of magnetization and bandgap obtained in the various samples with H_2_S, SO_2_ and SO_3_ adsorption on phosphorene of various metal decorations are summarized in Table 2. As mentioned above, a larger *E*_*ads*_ value above 1.5 eV is suitable for capturing gas molecules (or single sensing), whereas that near around 1 eV is ideal binding for highly efficient and reversible gas sensors. Therefore, from Table 1, one can see that the phosphorenes with AEM and La decorations can be served as capturing the SO_2_ and SO_3_ molecules, and that those with Li and Ni decorations can only be utilized as the SO_3_ capture. From Table 2, it is observed that the Li, Ca, Sr and Ba decorated phosphorenes can be used as selective single sensing for SO_3_ on the basis of increase of bandgap and thus decrease of conductivity after SO_3_ adsorption.

**Table 2:** The magnetic moment (*M*), bandgap (*E*_*g*_), and direct/indirect bandgap (D/I) of sulfur-based gas on phosphorene and bP-Ms.

Metal	bP-M			H_2_S-bP-M			SO_2_-bP-M			SO_3_-bP-M		
	*M*	*E*_*g*_	D/I	*M*	*E*_*g*_	D/I	*M*	*E*_*g*_	D/I	*M*	*E*_*g*_	D/I
Li	0	0		0	0		0.71	0		1.00	0.896/0.493	D/I
Na	0	0		0	0		0.58	0		0.92	0.094/0.839	I
K	0	0		0	0		0.50	0		0.81	0	
Rb	0	0		0	0		0.50	0		0.61	0	
Cs	0	0		0	0		− 0.46	0		0.76	0	
Ca	0	0.119	I	0	0.05	I	0.99	0		0	0.710	I
Sr	0	0.071	I	0	0		0.99	0		0	0.626	I
Ba	0	0		0	0		1.00	0		0	0.868	D
Ni	0	0.769	D	0	0.858	D	0	0.876	D	0	0.882	I
La	1.00	0.116/0.299	I	0.98	0/0.210	I	0	0		0	0	
Tl	0			0			0.51			0.99	0.417/0.738	I

The units of *M* and *E*_*g*_ are μ_B_ and eV, respectively.

The *E*_*ads*_ values of H_2_S on K, Rb, Cs and Tl decorated phosphorenes are so small that H_2_S gases are easy to dissociate from substrates, and thus the K, Rb, Cs and Tl decorated phosphorenes are not suitable for H_2_S sensing. In addition to the moderate *E*_*ads*_ value, the change in measurable property is necessary for reversible gas sensors. Therefore, the Tl decorated phosphorene is promising to be a selective reversible SO_3_ sensor due to the metal-to-semiconductor transition after gas molecule adsorption, while the semiconductor-to-half-metal transition of the La decorated phosphorene makes it be the potential candidate as a selective reversible H_2_S sensor. Interestingly, it is found that the magnetic moment turns positive and negative, respectively, after SO_2_ and SO_3_ adsorptions, which may be a basis of selectively sensing these gases.

## Conclusions

In summary, the adsorption of H_2_S, SO_2_ and SO_3_ on various metal-decorated phosphorene have been systematically investigated by using DFT. Eleven metals (Li, Na, K, Rb, Cs, Ca, Sr, Ba, Ni, La, Tl), *E*_ads_/*E*_coh_ > 1, are considered. Excepting H_2_S on bP-Tl, a decoration of metals has significantly improved the adsorption of phosphorene to sulfur-based gas molecules, and the order of adsorption capacity is La > AEM > AM. In the analysis of LDOSs, it is found that the phosphorenes with Sr and La decorations undergo the transitions from semiconductor to metal and from semiconductor to half-metal after H_2_S adsorption. The AM, AEM and Tl decorated phosphorenes undergo non-magnetic-to-magnetic transitions, after SO_2_ adsorption, while the La decorated phosphorene undergoes magnetic-to-non-magnetic transition. Meantime, the Ca, Sr and La decorated phosphorenes also have transition from semiconductor to metal after SO_2_ adsorption. For SO_3_ adsorption cases, the AM and Tl decorated phosphorenes obtain non-magnetic-to-magnetic transitions, while the La decorated phosphorene perceive the transition from magnetic to non-magnetic. The Li, Na and AEM decorated phosphorenes experience the transitions from metal to semiconductor, while the La decorated phosphorene experiences semiconductor-to-metal transition. On the basis of the criterion of adsorption energy around 1 eV and the changes in properties, the phosphorenes with La and Tl decorations are promising selective reversible sensors for H_2_S and SO_3_ detections, respectively. According to whether the magnetic moment is positive or negative, the Cs decorated phosphorene could be a potential selective reversible sensor for SO_3_ or SO_2_ detection.

## Supplementary Information

Supplementary Information.
