# Supplementary material for: Effect of metal decoration on sulfur-based gas molecules adsorption on phosphorene
Source: Sci Rep. 2021 Sep 13;11:18179. doi: 10.1038/s41598-021-97626-4 (PMC8438081; doi:10.1038/s41598-021-97626-4)
Supplement: Supplementary file 1 — Supplementary Information. [file 41598_2021_97626_MOESM1_ESM.pdf]

Supporting Information

Effect of metal decoration on sulfur-based gas molecules adsorption on phosphorene

Yonghu Wang[a], Shuangying Lei[a,*], Ran Gao[a], Xiaolong Sun[a], Jie Chen [a,*]

[a]Key Laboratory of Microelectromechanical Systems of the Ministry of Education, Southeast University, Nanjing 210096, China.

*Corresponding author. E-mail address: lsy@seu.edu.cn (S. Lei) , seuc@seu.edu.cn

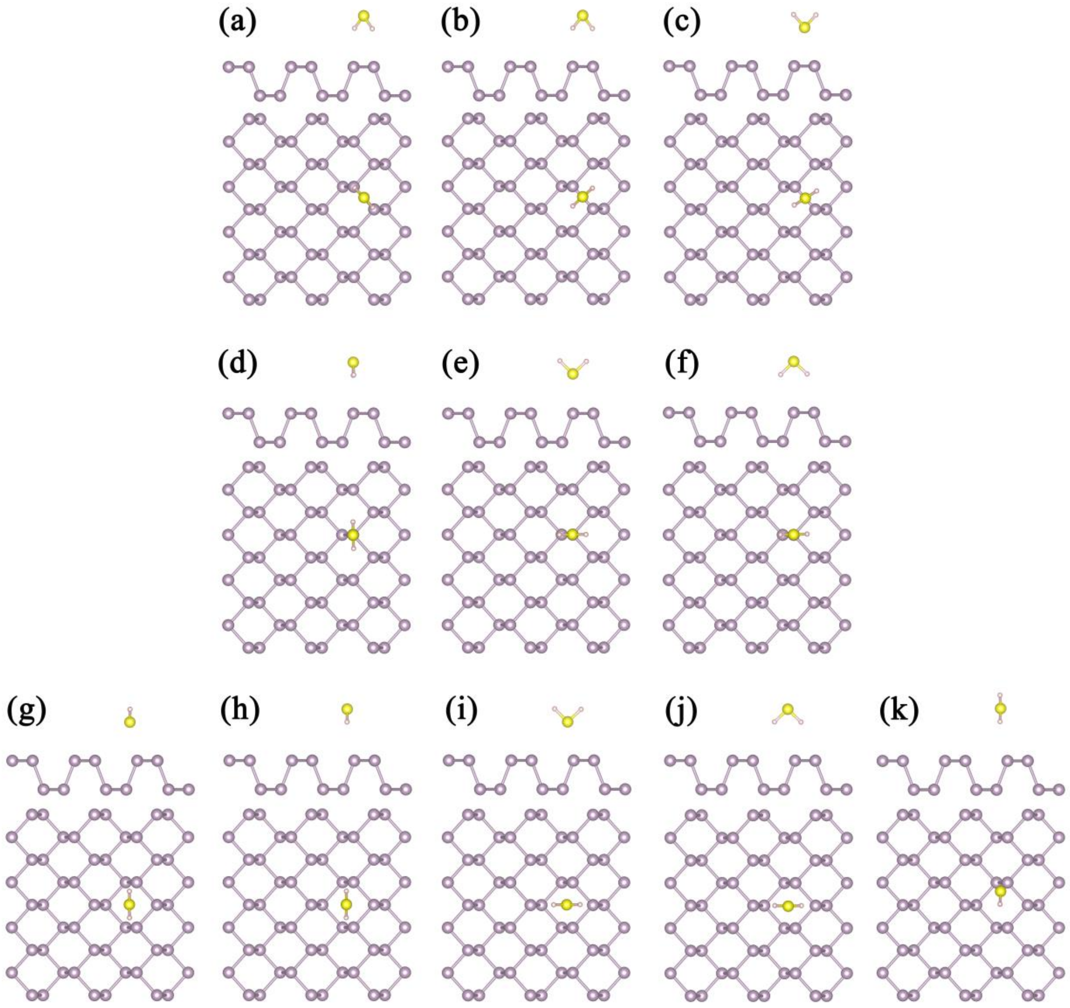

Figure S1. The initial structures of H_2S absorbed on 3×4 pristine phosphorene in different adsorption sites. (a)~(c) S atoms on bridge sites and H-H lines parallel to

phosphorene surface with S atoms towards different directions; (d)-(f) S atoms on top sites and H-H lines parallel to phosphorene surfaces with S atom towards different directions; (g)-(j) S atoms on hollow sites and H-H lines parallel to phosphorene surfaces with S atom towards different directions; (k) H-H line vertical to phosphorene surface. Purple, yellow and white balls represent P, S and H atoms, respectively.

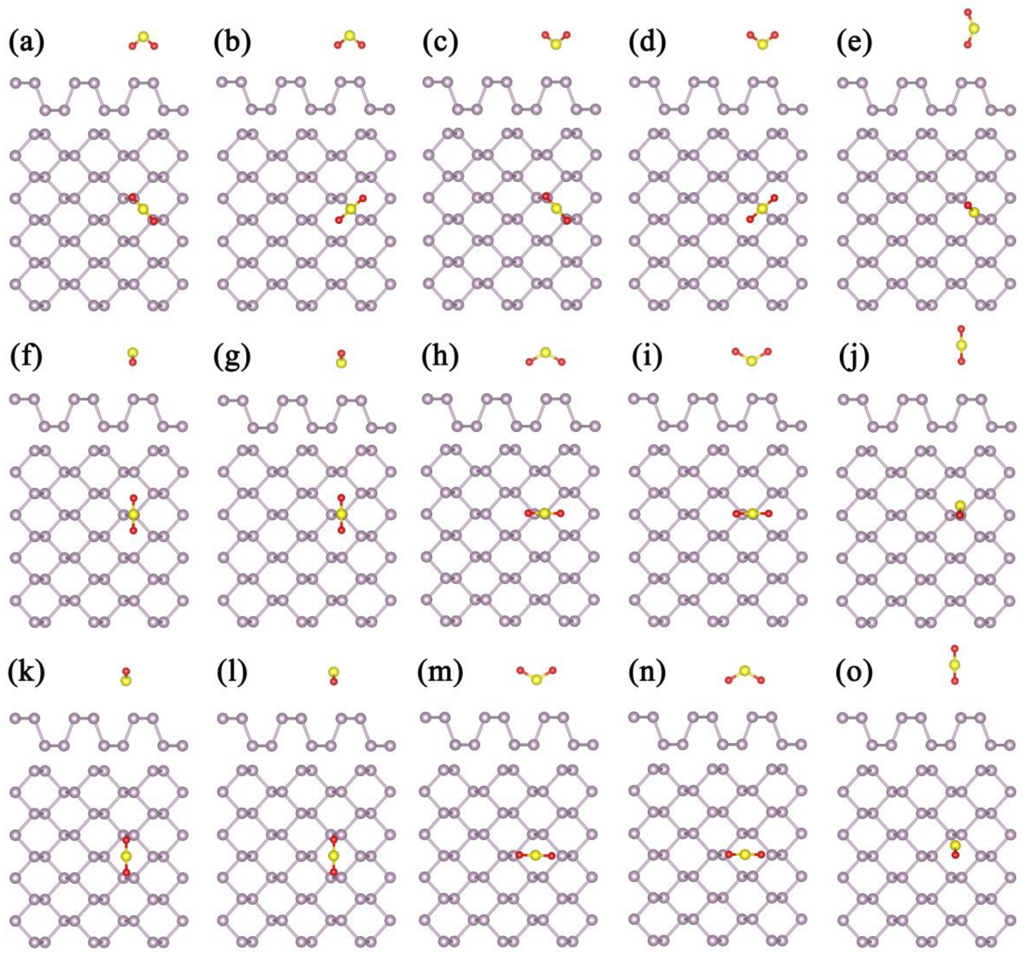

Figure S2. The initial structures of SO_2 absorbed on 3×4 pristine phosphorene in different adsorption sites. (a)-(d) S atoms on bridge sites and O-O lines parallel to phosphorene surfaces with S towards different directions; (f)-(i) S atoms on top sites and O-O lines parallel to phosphorene surfaces with S towards different directions; (k)-(n) S atoms on hollow sites and O-O lines parallel to phosphorene surfaces with S towards different directions; O-O lines vertical to phosphorene surfaces with O atoms on (e) bridge, (j) top and (o) hollow sites. Purple, yellow and red balls represent P, S, O atoms, respectively.

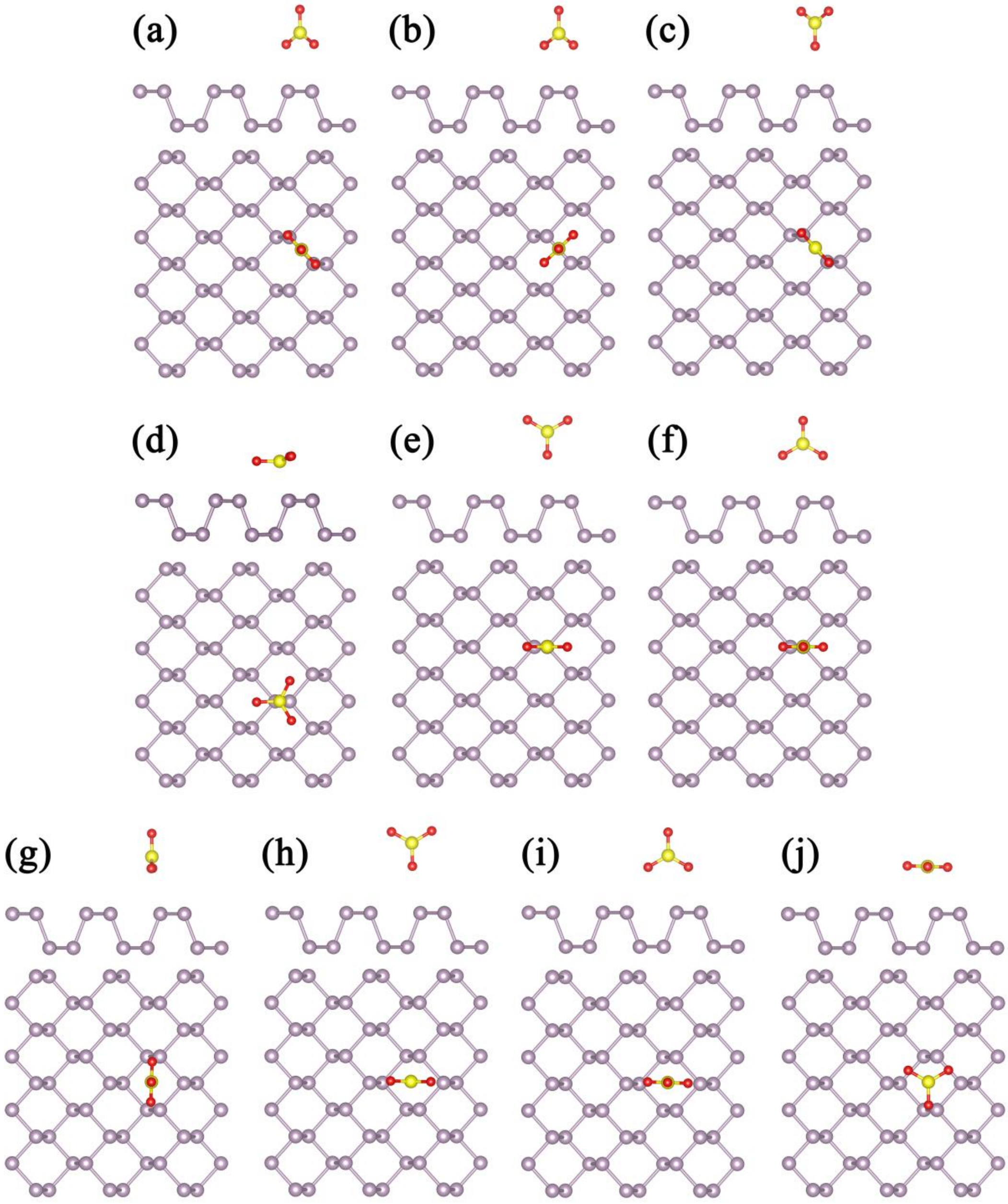

Figure S3. The initial structures of SO_3 absorbed on 3×4 pristine phosphorene in different adsorption sites. S atoms on bridge sites with O-S-O bond angle (a)-(b) toward phosphorene surface and (c) toward outside; S atoms on top sites with (d) O-atomic surface parallel to phosphorene surface, with O-S-O bond angle toward (e) outside and (f) phosphorene surface; S atoms on hollow sites with O-atomic surfaces (g)-(i) perpendicular and (j) parallel to phosphorene surfaces. Purple, yellow and red balls represent P, S, O atoms, respectively.

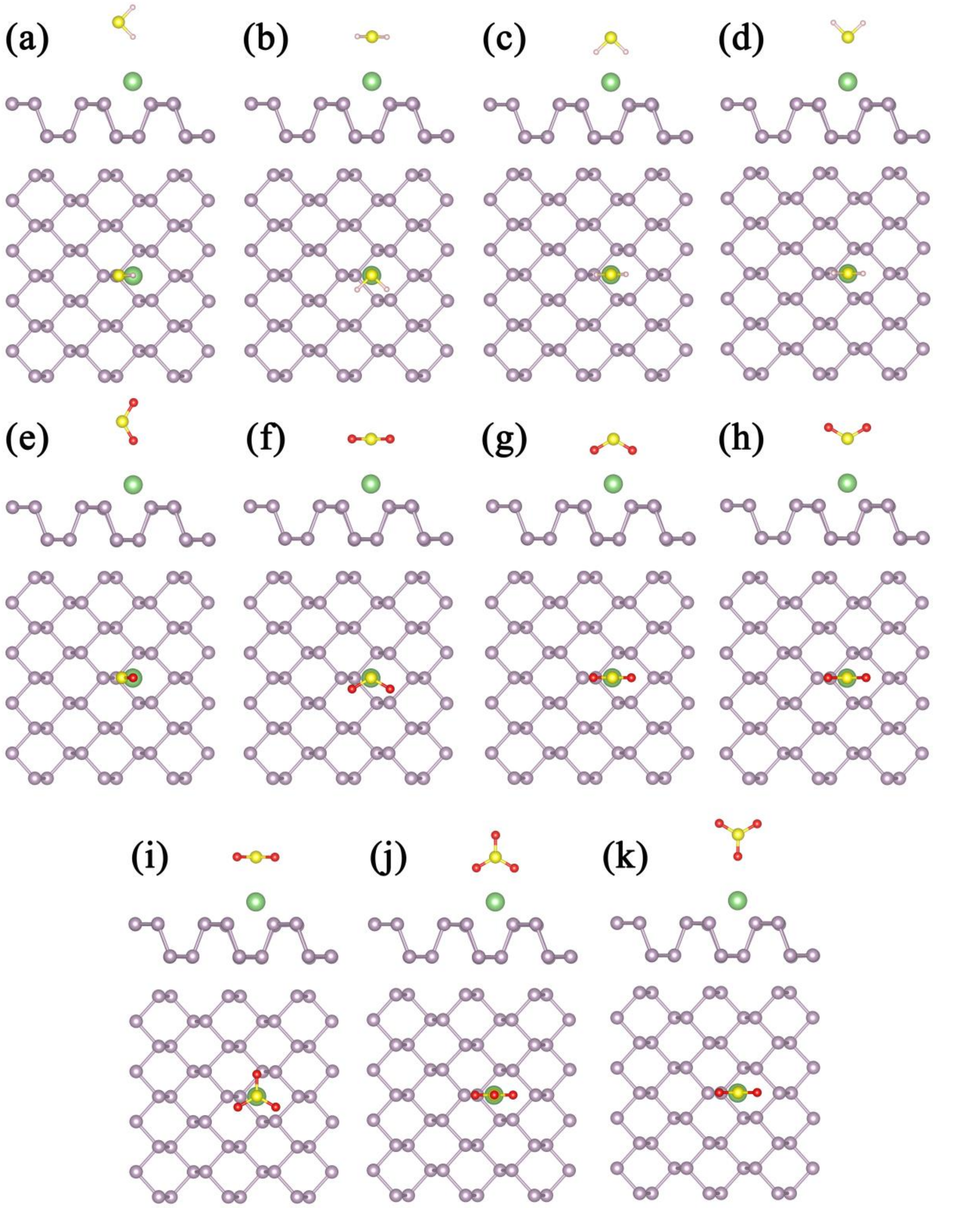

Figure S4. The initial structures of (a)-(d) H_2S, (e)-(h) SO_2, (i)-(k) SO_3 absorbed on Li doped phosphorenes. Purple, yellow, white, red and green balls represent P, S, H, O and Li atoms, respectively.

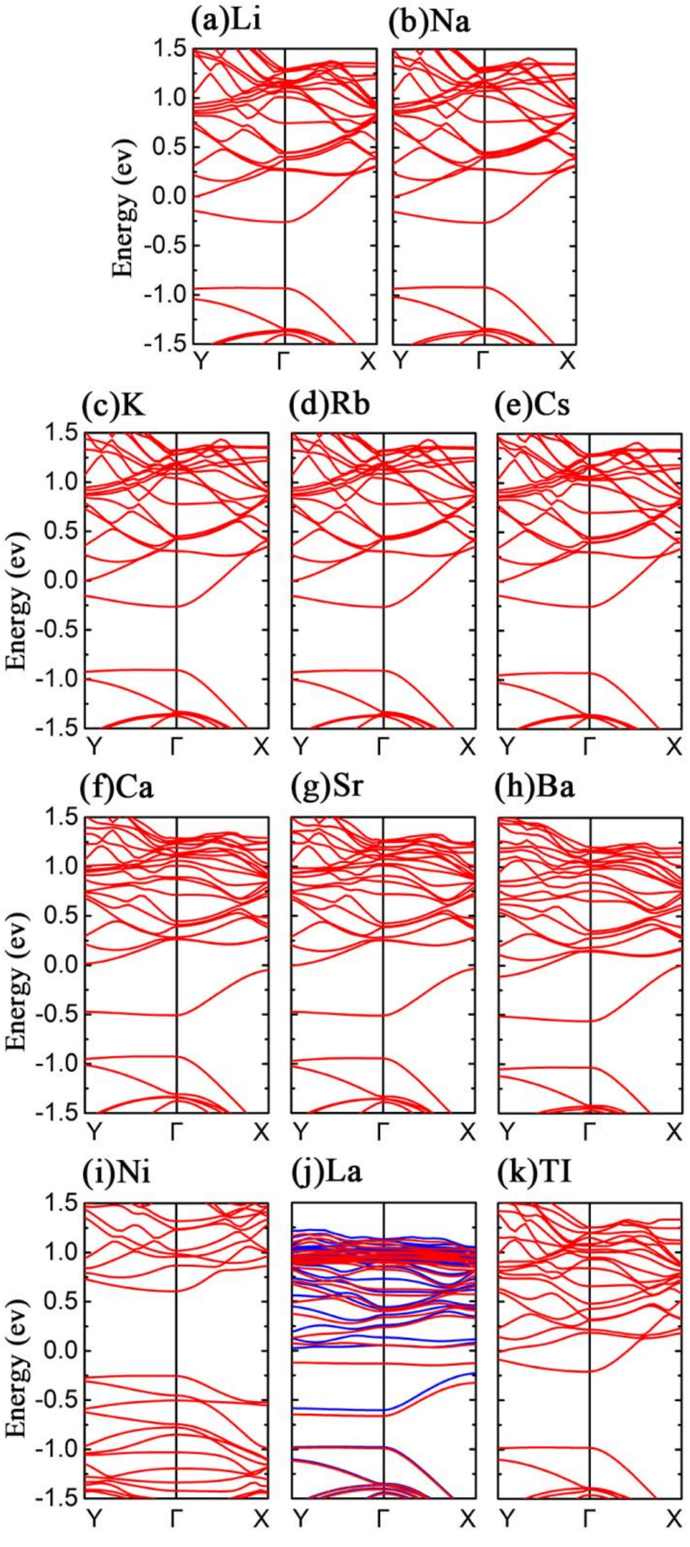

Figure S5. The band structures of H_2S adsorbed on (a) bP-Li, (b) bP-Na, (c) bP-K, (d) bP-Rb, (e) bP-Cs, (f) bP-Ca, (g) bP-Sr, (h) bP-Ba, (i) bP-Ni, (j) bP-La and (k) bP-Tl. The red and blue curves represent the spin-up and spin-down bands, respectively, with the Fermi level set to zero.

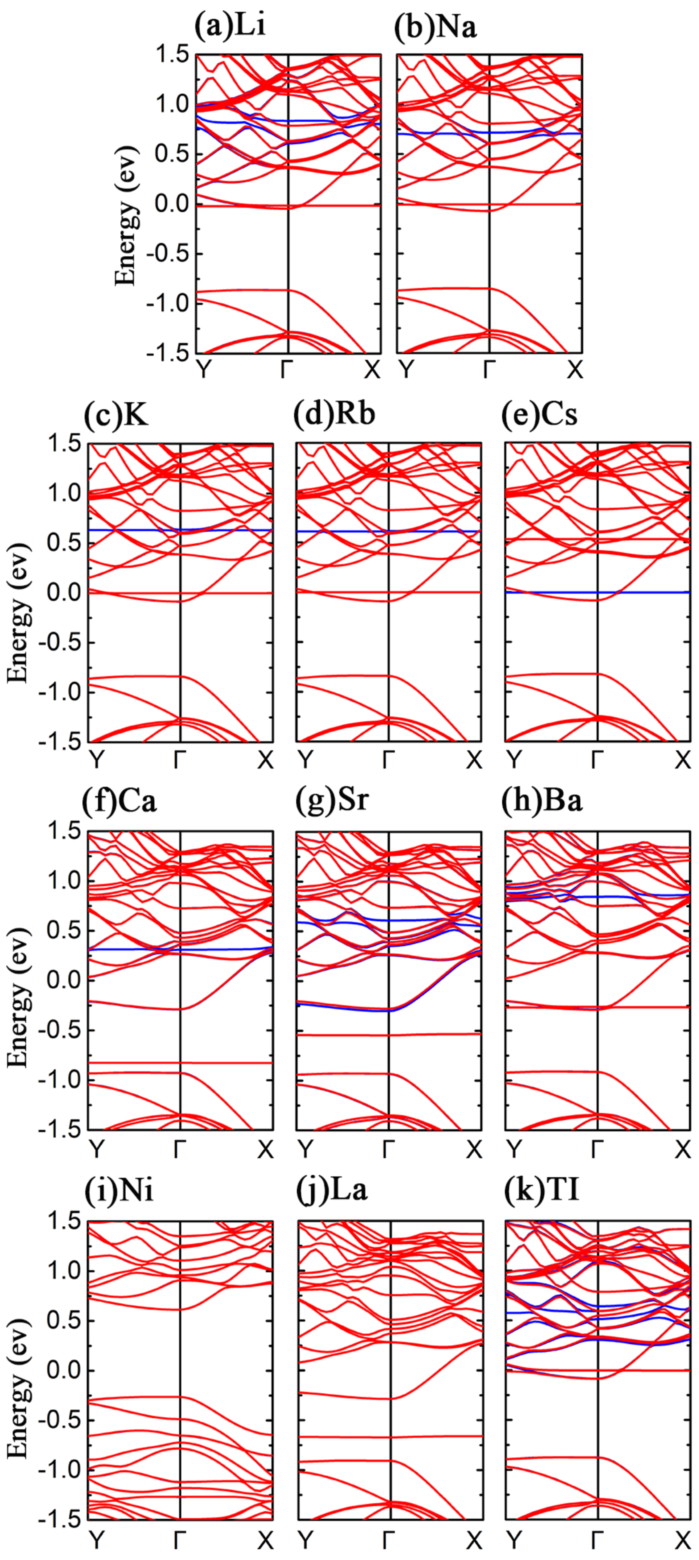

Figure S6. The band structures of SO_2 adsorbed on (a) bP-Li, (b) bP-Na, (c) bP-K, (d) bP-Rb, (e) bP-Cs, (f) bP-Ca, (g) bP-Sr, (h) bP-Ba, (i) bP-Ni, (j) bP-La and (k) bP-Tl. The red and blue curves represent the spin-up and spin-down bands, respectively, with the

Fermi level set to zero.

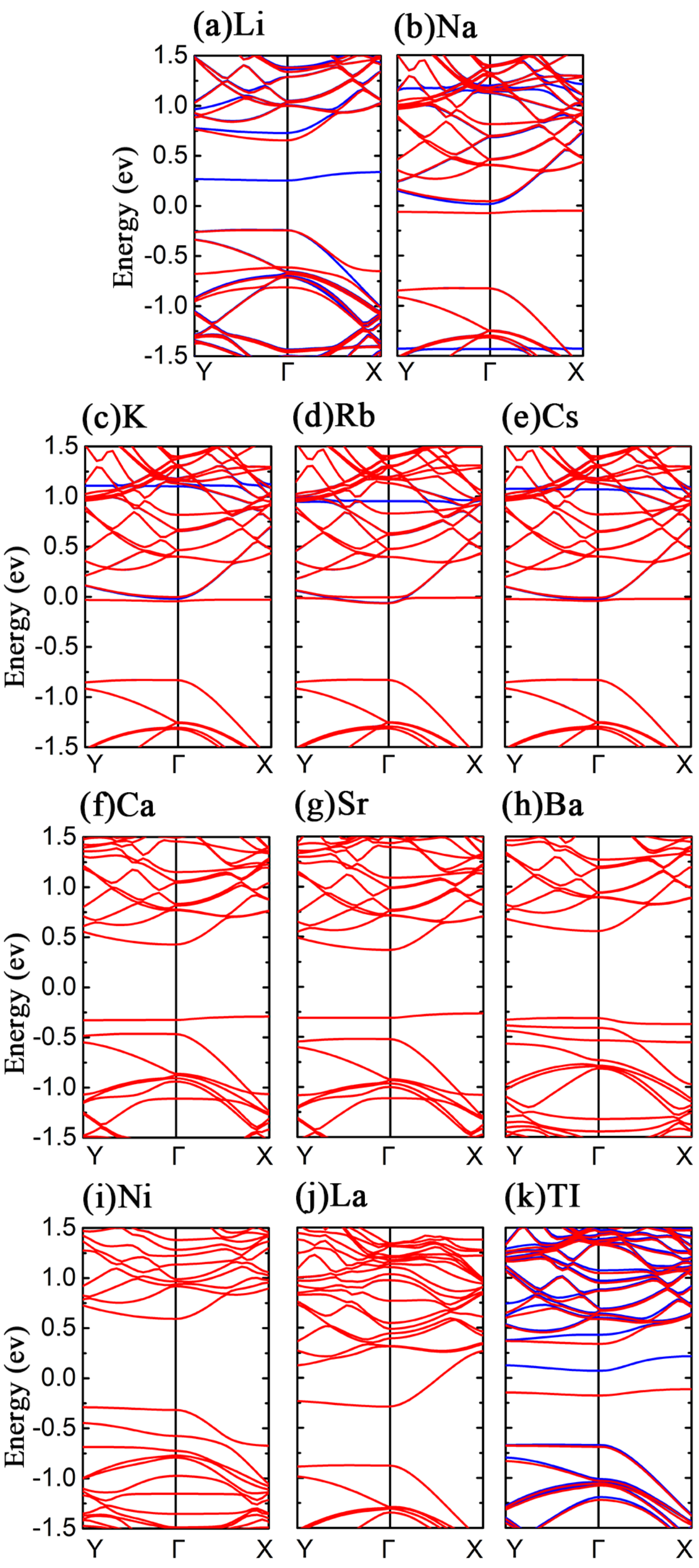

Figure S7. The band structures of SO_3 adsorbed on (a) bP-Li, (b) bP-Na, (c) bP-K, (d) bP-Rb, (e) bP-Cs, (f) bP-Ca, (g) bP-Sr, (h) bP-Ba, (i) bP-Ni, (j) bP-La and (k) bP-Tl. The red

and blue curves represent the spin-up and spin-down bands, respectively, with the Fermi level set to zero.
